# Graphene-Based Organic Semiconductor Film for Highly Selective Photocatalytic CO_2_ Reduction

**DOI:** 10.3390/nano15090677

**Published:** 2025-04-29

**Authors:** Yanghong Xu, Haopeng Tang, Yifei Wang, Xiaofeng Zhu, Long Yang

**Affiliations:** State Key Laboratory of Environment-Friendly Energy Materials, School of Materials and Chemistry, Southwest University of Science and Technology, Mianyang 621010, China; xuyanghong1024@163.com (Y.X.); thp88882025@163.com (H.T.); 13389317773@163.com (Y.W.)

**Keywords:** graphene, organic semiconductor, photocatalysis, CO_2_ reduction, 2D interface

## Abstract

Mimicking artificial photosynthesis utilizing solar energy for the production of high-value chemicals is a sustainable strategy to tackle the fossil fuel-based energy crisis and mitigate the greenhouse effect. In this study, we developed a two-dimensional (2D) graphene oxide (GO)–diketopyrrolopyrrole (DPP) film photocatalyst. GO nanosheets facilitate the uniform dispersion of DPP nanoparticles (~5 nm) while simultaneously constructing an efficient charge transport network to mitigate carrier recombination. Under visible-light irradiation in an aqueous solution without sacrificial agents, the optimized GO–DPP50 film catalyst exhibited exceptional performance, achieving a CO production rate of 32.62 μmol·g⁻^1^·h⁻^1^ with nearly 100% selectivity. This represents 2.77-fold and 3.28-fold enhancements over pristine GO (8.65 μmol·g^−1^·h^−1^) and bare DPP (7.62 μmol·g^−1^·h^−1^), respectively. Mechanistic analysis reveals a synergistic mechanism. The 2D GO framework not only serves as a high-surface-area substrate for DPP anchoring, but also substantially suppresses charge recombination through rapid electron transport channels. Concurrently, the uniformly distributed DPP nanoparticles improve visible-light absorption efficiency and facilitate effective photogenerated carrier excitation. This work establishes a novel paradigm for the synergistic integration of 2D nanomaterials with organic semiconductors, providing critical design principles for developing high-performance film-based photocatalysts and selectivity control in CO_2_ reduction applications.

## 1. Introduction

The 21st century has witnessed an unprecedented surge in global energy consumption, with fossil fuels still dominating over 85% of the world’s primary energy supply. This persistent reliance on fossil resources has triggered a dramatic escalation in atmospheric carbon dioxide (CO_2_) concentrations, which have soared from preindustrial levels of 280 parts per million (ppm) to the current critical threshold exceeding 420 ppm [1]. As the most prevalent anthropogenic greenhouse gas, CO_2_ has emerged as the central driver of climate change, intensifying challenges such as global warming, ocean acidification, and the increasing frequency of extreme weather events [2,3,4]. In response, the international community has adopted ambitious emission reduction targets and is vigorously advancing carbon capture, utilization, and storage (CCUS) technologies as core components of its carbon neutrality agendas [5,6].

Amid this technological landscape, CO_2_ conversion and reduction technologies have garnered worldwide attention for their dual potential to alleviate energy security risks and environmental degradation. These technologies are designed not only to reduce CO_2_ emissions in the atmosphere, but also to convert this greenhouse gas into valuable chemicals and fuels through catalytic processes, thereby supporting a circular economy model that promotes resource recycling [7,8]. Among these solutions, photocatalytic CO_2_ reduction stands out as a particularly promising approach due to its operation under mild conditions, minimal environmental footprint, and capacity to directly harness solar energy for synthesizing high-value chemical products. The photocatalytic mechanism operates through a series of photon-driven redox reactions: when illuminated by light with appropriate energy, electrons in the valence band of a photocatalyst are excited to the conduction band, generating electron–hole pair carriers. These photogenerated charge carriers transfer to the active sites on the catalyst surface, where electrons drive the reduction of CO_2_ molecules into valuable chemical products (e.g., HCOOH, CO, or CH_4_). Concurrently, holes engage in the oxidation of water, generating molecular oxygen as a byproduct [9,10,11]. This process effectively bridges renewable energy conversion and carbon fixation, offering a sustainable pathway to close the anthropogenic carbon cycle by utilizing sunlight—an abundant and renewable resource—to convert waste CO_2_ into energy-dense fuels.

However, photocatalytic CO_2_ conversion currently struggles with low efficiency and poor product selectivity. To address this challenge, two-dimensional (2D) materials are being explored for their potential to solve the related issue. Their high surface area, abundant active sites, and efficient charge/proton transport properties make them ideal candidates for development in this area [12,13]. Among them, graphene-based materials excel in conductivity, stability, and structural flexibility. Additionally, organic semiconductors, with adjustable electronic properties and functional groups, could enhance CO_2_ adsorption and improve product control [14,15,16]. Consequently, combining graphene with semiconductors will boost charge separation and reaction efficiency, enhancing overall performance [17,18,19]. Moreover, film photocatalysts offer superior practicality for real-world applications by addressing the key challenges of powdered systems—recovery, stability, scalability, secondary pollution, light utilization, and environmental safety—while enabling advanced reactor designs and functional architectures [20,21,22].

Hence, guided by our previous research [23,24,25,26], in this work, a graphene-based organic semiconductor material and a film photocatalyst (GO–DPP) was successfully constructed. The DPP molecule was rationally screened due to the unique functional group of N-unsubstituted amide and the derived striking performance in CO_2_ activation [27]. The composite’s structural, chemical, and optical properties were comprehensively characterized, and its photocatalytic performance for CO_2_ reduction was systematically evaluated. These results elucidate the function of 2D interfaces in enhancing charge dynamics and catalytic performance, thereby providing valuable insights for the rational design of advanced graphene-based photocatalysts.

## 2. Experimental Sections

### 2.1. Materials

**Figure 1:**
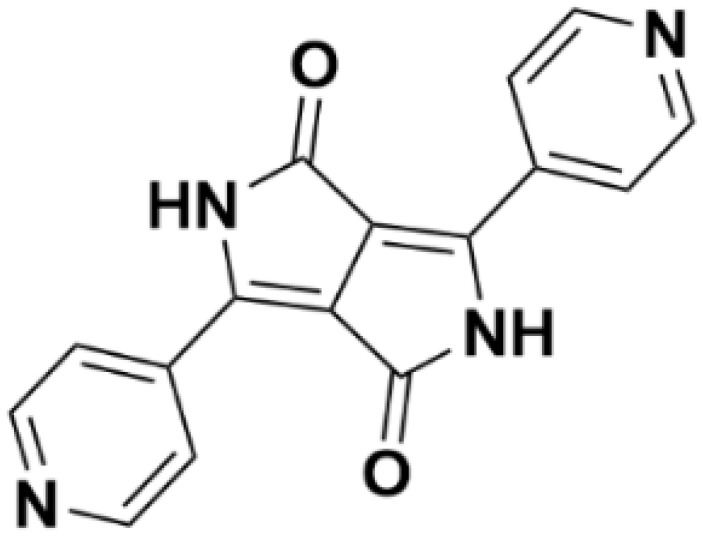
The molecular structure of DPP.

All reagents were commercially available and used as received without further purification. NaOH (AR), ethanol (99.99%), HCl (99.99%), GO (98%), and DPP (AR) were purchased from Yuanye Bio-Technology Co., Ltd. (Shanghai, China). Ultrapure water was prepared using a Millipore system (18.25 MΩ·cm, Billerica, MA, USA). The structural formula of the DPP molecule is shown in Figure 1.

### 2.2. Material Synthesis

**Scheme 1:**
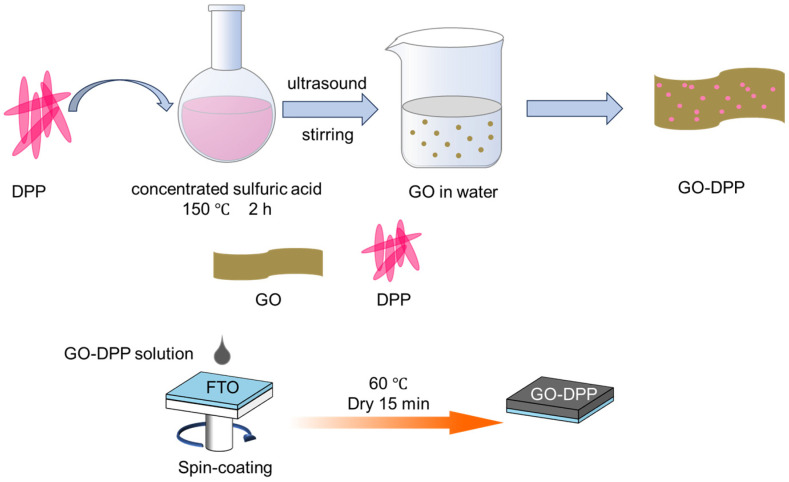
Schematics on the preparation of a composite GO–DPP film catalyst.

The synthesis method is shown in Scheme 1. Initially, 50 mg of DPP were dissolved in 5 mL of concentrated sulfuric acid. This solution was heated to 150 °C under magnetic stirring and maintained at this temperature for 2 h to form a uniform mixture. Simultaneously, 100 mg of GO were dispersed in 100 mL of ultrapure water through sonication for 1 h, resulting in a stable colloidal suspension. The prepared DPP solution was then added dropwise at a slow rate to the GO dispersion while stirring continuously. After the addition, the mixture was stirred for an additional 30 min. Subsequently, the resulting material was filtered and subjected to sequential washing with 0.1 M NaOH, 0.1 M HCl, and deionized water to eliminate impurities. The purified product underwent freeze-drying for 24 h, yielding the GO–DPP50 composite. For comparative analysis, another composite with a DPP/GO mass ratio of 20 mg:100 mg (designated as GO–DPP20) was synthesized using an identical procedure.

The catalyst dispersion process began by dispersing 10 mg of the catalyst into a mixed solvent composed of 2.5 mL ultrapure water and 2.5 mL ethanol. The mixture underwent ultrasonic treatment for 30 min to ensure uniform distribution. A fluorine-doped tin oxide (FTO) glass substrate, measuring 1.5 cm × 2 cm, was chosen as the support material for film deposition. Using a spin-coating technique, 50 μL aliquots of the prepared catalyst dispersion were applied sequentially to the FTO surface. Each coating cycle involved spinning the substrate at 800 revolutions per minute for 15 s, and this procedure was repeated 20 times to build up the film layer. After spin-coating, the coated substrate was transferred to an oven set at 60 °C and dried for 15 min to form the final film catalyst.

### 2.3. Characterizations

X-ray diffraction (XRD) enables the characterization of material properties, including crystalline structure, crystal plane orientation, lattice parameters, and interlayer spacing. Fourier-transform infrared spectroscopy (FT-IR) analysis is used to investigate the structure and chemical bonds of catalyst materials. X-ray photoelectron spectroscopy (XPS) is used to precisely characterize information regarding molecular structure and atomic valence states. Additionally, it provides critical information for electron material research, such as elemental composition, chemical states, and molecular structure. CO_2_ temperature-programmed desorption (CO_2_-TPD) is employed to investigate the interaction mechanism between catalyst surfaces and CO_2_ molecules. By precisely measuring the variation in desorbed CO_2_ amounts with temperature during a programmed heating process, this technique elucidates critical information about the acid–base properties, active site distribution, and adsorption strength of the sample surface. Electrochemical impedance spectroscopy (EIS) is mainly used to study the separation efficiency of photogenerated charges, interfacial charge transfer characteristics, and carrier dynamics behavior of catalysts.

### 2.4. Photocatalytic Reduction Experiment

**Scheme 2:**
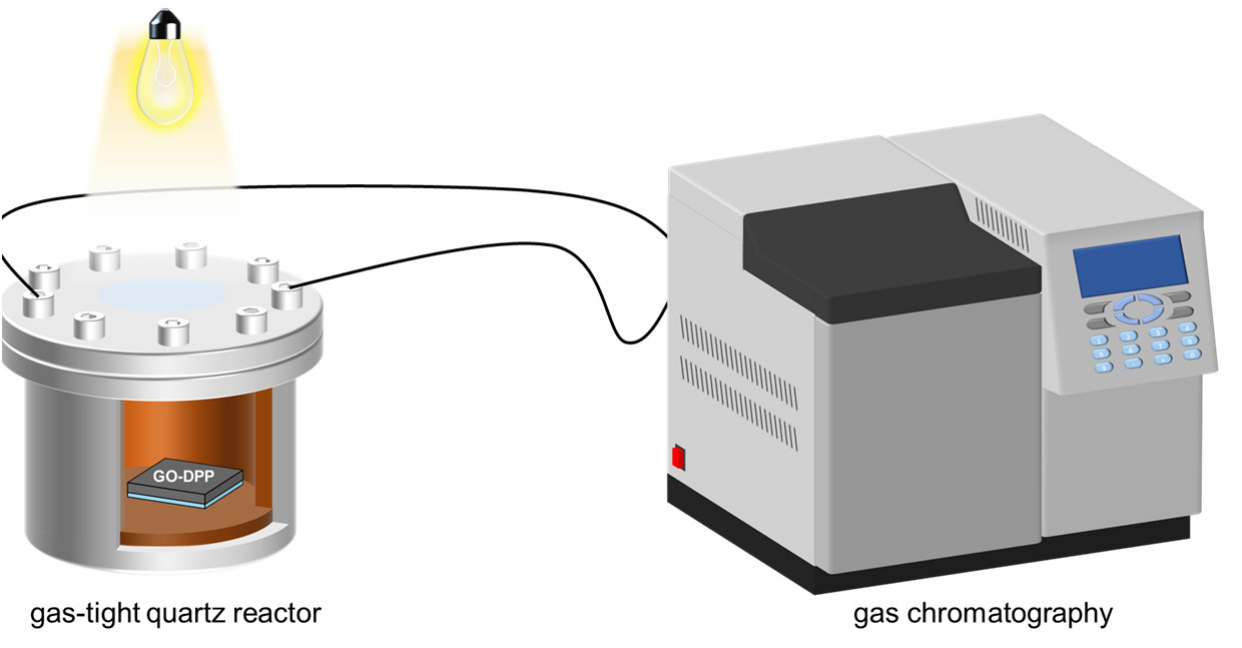
Schematic diagram of the experimental setup.

The experimental setup is shown in Scheme 2. To eliminate potential CO contamination from GO photolysis, all the samples underwent a critical pretreatment step: 5 h of visible-light irradiation under a nitrogen atmosphere to ensure complete decomposition of residual carbonaceous species. Catalytic performance testing was then conducted using a commercial photocatalytic evaluation system featuring a quartz reactor cell operating in the vapor–solid reaction mode. Specifically, the film catalyst was positioned at the center of the reactor. Next, 200 μL of ultrapure water were introduced as a proton source. The system was sequentially sealed, evacuated to <0.1 kPa, and backfilled with high-purity CO_2_ to achieve a reaction pressure of 200 kPa. Photocatalytic CO_2_ reduction was initiated using a 300 W Xe lamp (light intensity: 280 mW·cm^−2^) fitted with a 400 nm cutoff filter to simulate solar irradiation. Product quantification was performed via gas chromatography (GC-7890B, Agilent Technologies, Flanders, NJ, USA) using argon a s the carrier gas, with the column temperature maintained at 50 °C. Control experiments confirmed that no detectable CO formation occurred in the absence of CO_2_, even after prolonged reaction times. Furthermore, negligible product yield was observed under dark conditions, confirming the reaction’s exclusive dependence on visible light activation. In addition, the post-reaction liquid was collected and subjected to ^1^H nuclear magnetic resonance (^1^H-NMR) spectroscopy analysis to verify the absence of any potential liquid-phase products (e.g., formic acid).

## 3. Results and Discussion

### 3.1. Morphological and Structural Characterization

**Figure 2:**
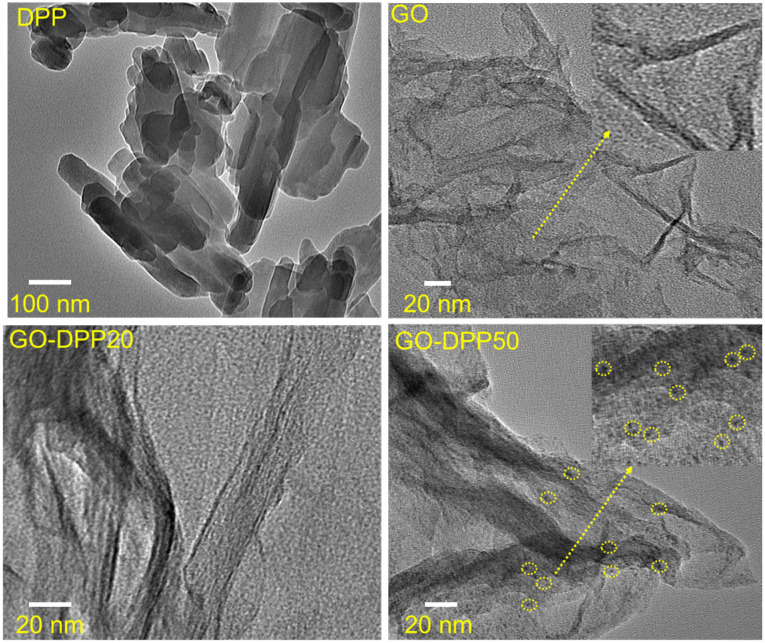
TEM images of DPP, GO, and composite GO–DPP.

As shown in Figure 2, the TEM characterization revealed distinct morphological evolution and interfacial interactions in the composite system. In the TEM image of DPP, large-sized crystalline particles dominated by bulk or rod-like structures are observed, exhibiting a high degree of aggregation. In contrast, GO displays a nanoscale wrinkled structure with a transparent flake morphology and high-contrast edges. The surface of the material features oxygen-containing functional groups, such as hydroxyl and epoxy groups, which serve as active sites for the composite [28,29]. In the TEM image of GO–DPP50, under a 20 nm scale, DPP crystalline particles are clearly observed to be uniformly dispersed on the 2D sheets of GO. Compared to DPP, the particle size of DPP crystals in GO–DPP50 (about 5 nm) is significantly reduced, indicating that the layered structure of GO effectively inhibits the growth and aggregation of DPP crystals during the composite process. The morphology of GO–DPP20 is similar to that of GO, and no distinct DPP particles were observed.

**Figure 3:**
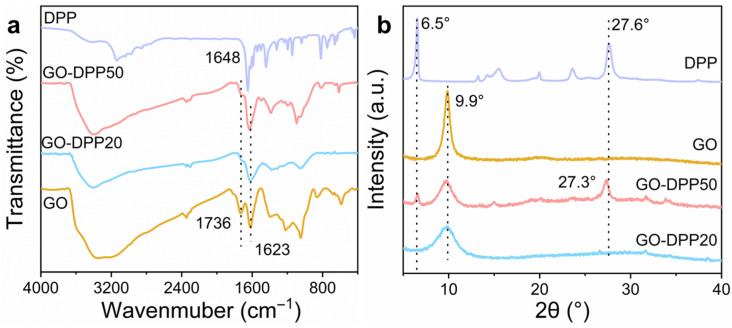
FT-IR spectra (**a**) and XRD patterns (**b**) of DPP, GO, and composite GO–DPP.

Next, the compositional structure of GO–DPP was investigated through FT-IR, XRD, and XPS. In the FT-IR spectra (Figure 3a), for the infrared spectrum of GO, the absorption peaks at 1623 cm^−1^ and 1736 cm^−1^ correspond to the stretching vibrations of C=C and C=O bonds, respectively. Meanwhile, the broad peak in the 3200–3600 cm^−1^ region indicates the presence of extensive hydrogen bonding. As for DPP, due to the conjugation between the lone pair electrons of the N atom in the pyrrole ring and the adjacent unsaturated C=O bond, a mesomeric effect occurs [30,31]. The complete disappearance of the graphite peak near 26° indicates full oxidation. For DPP, characteristic diffraction peaks are observed at 6.5° and 27.6°. The GO–DPP50 composite retains the characteristic diffraction peaks of DPP intact, with the 27.6° peak shifted leftward to 27.3°, suggesting an increased interlayer spacing. Additionally, a weak broad diffraction peak near 26° indicates that partial reduction of GO occurred during the composite formation [32,33]. Moreover, GO–DPP20 exhibited no distinct characteristic diffraction peaks of DPP, except for the feature at 9.9° corresponding to GO. Additionally, the change in interlayer spacing of the GO–DPP composite can be calculated using the Bragg equation. The diffraction peak of GO–DPP at 27.3° corresponds to an interlayer spacing of 3.26 Å, while the diffraction peak of DPP at 27.6° corresponds to an interlayer spacing of 3.23 Å. After the GO and DPP composite forms, DPP molecules are inserted into the interlayer of GO, resulting in an increase in the interlayer spacing [34,35].

**Figure 4:**
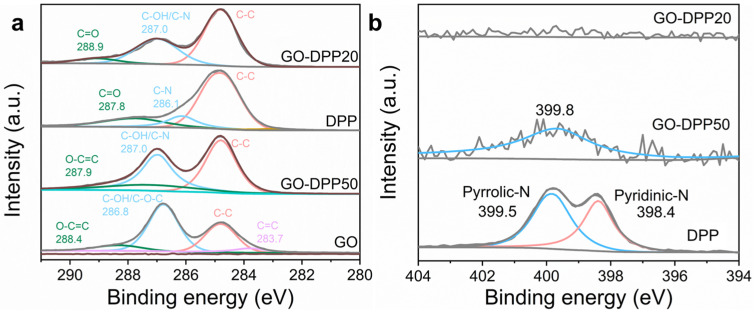
XPS spectra ((**a**) C1s; (**b**) N1s) of DPP, GO, and composite GO–DPP.

Regarding the elemental composition, the XPS survey spectra of all the samples, as shown in Figure 4a, exhibited C1s signals. The XPS C1s spectra of GO were deconvoluted into four individual peaks as follows: sp^2^ C=C at 283.7 eV, sp^3^ C–C at 284.8 eV, C–OH/C–O–C at 286.8 eV, and O–C=C at 288.4 eV. Analyzing the XPS C1s spectra of GO–DPP50 after deconvolution, the spectrum is divided into several characteristic peaks [36]. The 284.8 eV peak corresponds to C–C in the carbon skeleton, the 287.0 eV peak—to C–OH/C–N. The 288.4 eV peak (O–C=O) retains GO’s carbonyl-like oxygen-containing functional group characteristics. For GO–DPP20, the binding energies at 287.0 eV and 288.9 eV correspond to C–OH/C–N and C=O bonds, respectively [37,38]. Figure 4b presents the N1s spectrum, where GO–DPP20 exhibited no distinct N1s signals. Combined with the above FT-IR and XRD results, this suggests that the DPP content might be too low for detection.

Overall, DPP and GO establish tight 2D interfacial bonding through π–π stacking and van der Waals forces. Upon compounding with GO, DPP disperses uniformly on the 2D GO platform, remarkably reducing crystal aggregation. The bulk structure evolves from original large-sized (100 nm × 300 nm) rod-like crystals into structures with a smaller size of approximately 5 nm. These results demonstrate the successful synthesis of GO–DPP.

### 3.2. Optical Properties and Catalytic Performance

**Figure 5:**
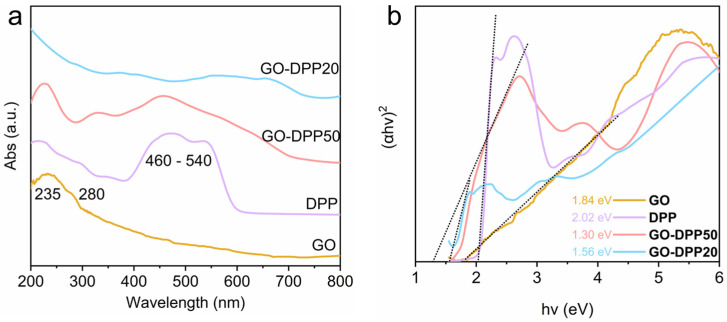
The DRS UV–Vis (**a**) and Tauc plots (**b**) of DPP, GO, and composite GO–DPP.

To explore the electronic structure of GO–DPP, UV–vis diffuse reflectance spectroscopy (DRS) was employed to characterize the absorption properties of GO, DPP, and GO–DPP. As depicted in Figure 5a, GO exhibited a strong absorption peak at 235 nm, which arose from the π–π transition of its sp^2^-conjugated structure. The shoulder peak observed at 280 nm resulted from the π–π transition of oxygen-containing functional groups attached to the GO framework. For DPP, a broad absorption band spanning 460–540 nm can be ascribed to the π–π* transition within its intramolecular conjugated structure. Notably, compared with pure GO and DPP, the absorption edge of GO–DPP50 displays a pronounced redshift, indicating an expanded spectral range for light absorption. This shift signifies a decrease in the energy barrier for electron transitions, enabling the material to absorb lower-energy photons more efficiently. Such a property enhances light-harvesting capacity and generates additional photo-induced charge carriers, which are critical for improving catalytic performance [39,40]. Figure 5b presents the Tauc plots, from which the band gap energy (E_g_) was derived via conversion of UV–vis DRS data using the Kubelka–Munk function. The E_g_ values for GO, DPP, GO–DPP50, and GO–DPP20 were determined as 1.84 eV, 2.02 eV, 1.30 eV, and 1.56 eV, respectively.

**Figure 6:**
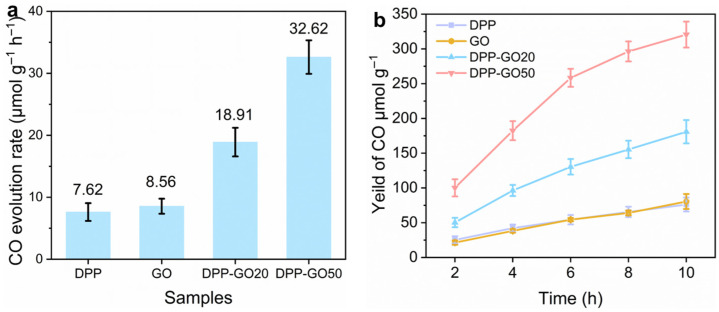
Comparison of the photocatalytic CO_2_ reduction performance to CO of GO, DPP, and composite GO–DPP.

Under irradiation with visible light (λ > 400 nm), the photocatalytic CO_2_ reduction performance of the samples was evaluated without incorporating any sacrificial agents. As shown in Figure 6, DPP50 demonstrated the highest photocatalytic CO_2_ reduction efficiency, approximately 32.67 μmol g^−1^ h^−1^, which is 3.82 and 4.23 times greater than that of GO and DPP, respectively. This suggests that the introduction of DPP50 significantly enhances the photocatalytic CO_2_ reduction process. Notably, DPP50 also exhibited excellent stability over a period of 10 h, demonstrating its long-term efficiency for CO_2_ reduction. In addition to its high efficiency, DPP50 showed remarkable 100% selectivity for CO production, which is a critical aspect in the photocatalytic reduction of CO_2_. This high selectivity demonstrates that the photocatalyst preferentially promotes CO formation over other potential byproducts (H_2_), underscoring the superior photocatalytic CO_2_ reduction performance of GO–DPP50.

**Figure 7:**
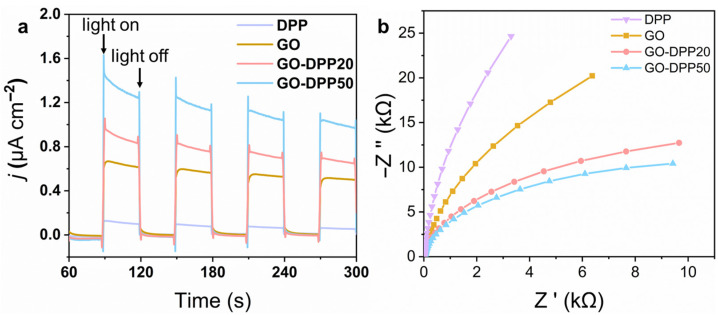
The transient photocurrent density (**a**) and electrochemical impedance spectroscopy (**b**) of GO, DPP, and composite GO–DPP.

The efficiency of separation and migration for photogenerated carriers represents a crucial factor governing photocatalytic performance [41,42]. The charge dynamics of GO–DPP composites were systematically evaluated using transient photocurrent density measurements. As depicted in Figure 7a, all GO–DPP samples exhibited significantly enhanced photocurrent responses compared to pristine GO and DPP. The photocurrent response of DPP was relatively low (~0.11 μA cm^−2^), reflecting the limited charge transport capability of its single-component system. Notably, the GO–DPP50 formulation achieved the highest photocurrent density (~1.3 μA cm^−2^), underscoring its superior charge separation efficiency. Furthermore, as shown in Figure 7b, the relatively high charge transfer resistance of pristine GO and DPP indicates low carrier migration efficiency and significant impedance to interfacial electron transfer. In stark contrast, the GO–DPP50 composite exhibited a drastic reduction in charge transfer resistance. This notable decrease is attributed to efficient charge transfer at the GO/DPP interface, where photoexcited electrons in DPP can readily migrate to CO_2_ molecules through the conductive π-network of GO [43,44,45].

**Figure 8:**
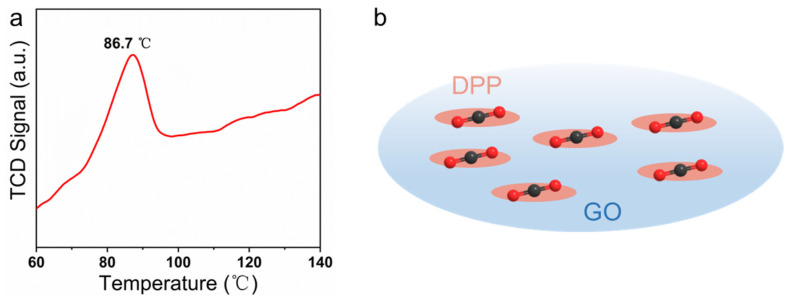
CO_2_-TPD profiles of DPP (**a**) and a diagram of DPP serving as CO_2_ adsorption and reaction sites in GO–DPP (**b**).

**Figure 9:**
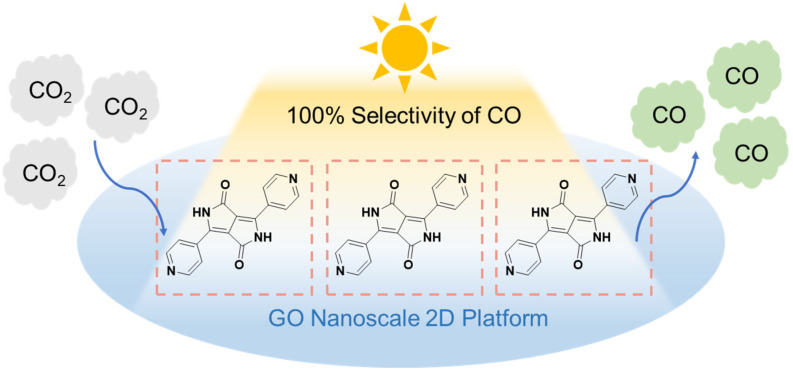
Highly selective photocatalytic CO_2_ reduction to CO over GO–DPP.

In addition, the photocatalytic CO_2_ reduction performance is largely dependent on the catalyst’s ability to adsorb CO_2_ effectively. The chemical adsorption of CO_2_ on the catalyst materials was characterized using CO_2_ temperature-programmed desorption (CO_2_-TPD). A distinct desorption signal for DPP was observed at 86.7 °C, confirming that DPP exhibited chemical adsorption of CO_2_ (Figure 8). This result indicates that DPP plays a crucial role in the adsorption of CO_2_, acting as both an adsorption and reaction site. The DPP component was uniformly dispersed onto the two-dimensional GO platform, which further enhanced the catalyst’s ability to adsorb CO_2_ molecules, ensuring a higher concentration of CO_2_ at the catalytic sites [46,47,48]. As shown in Figure 9, this GO–DPP composite photocatalyst demonstrates three key advantages: excellent carrier separation efficiency, enhanced CO_2_ adsorption capacity, and the ability to serve as an efficient electron transfer platform. The GO substrate, with its high conductivity and large surface area, facilitates the effective transport of electrons to the DPP sites. These combined properties—optimized charge carrier dynamics, improved reactant adsorption, and efficient electron transfer—enable the selective photocatalytic reduction of CO_2_ to CO under mild conditions without any sacrificial agents.

## 4. Conclusions

A graphene oxide-based GO–DPP composite photocatalyst was successfully synthesized through π–π stacking and van der Waals interactions, ensuring uniform dispersion of diketopyrrolopyrrole (DPP) nanoparticles on the two-dimensional GO substrate. The optimized GO–DPP50 catalyst demonstrated excellent photocatalytic CO_2_-to-CO conversion performance under visible-light irradiation, achieving a CO evolution rate of 32.62 μmol g^−1^ h^−1^ without the use of sacrificial agents. Mechanistic studies reveal that the conductive GO framework facilitates efficient electron transfer, while effectively suppressing charge recombination. This enhances the photocatalytic process by maintaining a high separation efficiency of photogenerated charges. Additionally, the nitrogen-enriched DPP component boosts CO_2_ chemisorption through Lewis acid–base interactions, facilitating the activation of CO_2_ molecules. The synergistic combination of optimized charge carrier dynamics and enhanced reactant adsorption significantly improves the photocatalytic efficiency. The study highlights the importance of dual optimization, focusing on both the electronic structure and surface chemistry of materials for sustainable carbon conversion. This work provides valuable insights into the design of advanced 2D nanomaterial-based photocatalysts, contributing to future research and development in clean energy technologies for efficient CO_2_ reduction under visible light.

## Data Availability

Data are contained within the article.
